# Old Plants for New Food Products? The Diachronic Human Ecology of Wild Herbs in the Western Alps

**DOI:** 10.3390/plants14010122

**Published:** 2025-01-03

**Authors:** Mousaab Alrhmoun, Aurora Romano, Naji Sulaiman, Andrea Pieroni

**Affiliations:** 1 University of Gastronomic Sciences, Piazza Vittorio Emanuele II 9, 12042 Pollenzo, Italy; mousaab.alrhmoun@unibz.it (M.A.); a.romano@studenti.unisg.it (A.R.); a.pieroni@unisg.it (A.P.); 2 Faculty of Agricultural, Environmental and Food Sciences, Free University of Bolzano, Piazza Università 5, 39100 Bolzano, Italy; 3 Department of Medical Analysis, Tishk International University, Erbil 4001, Iraq

**Keywords:** Bellino Valley, cross-border analysis, ethnobotany, ethnoecology, medicinal plants, traditional knowledge, Ubaye Valley, wild food plants

## Abstract

This ethnobotanical study examines the traditional knowledge and usage patterns of wild plants in the western Alps, specifically within the Ubaye and Bellino Valleys, through a comparative analysis of data collected from 1983 (published in 1990) to 2024. Our study aims to assess the change in plant usage, species diversity, and the changing roles of plants in local traditions in the western Alpine mountain ecosystems. While the 1983 survey documented medicinal uses centered around pastoralist practices, the 2024 data highlight a notable increase in the use of synanthropic plants, now utilized both medicinally and as food. Several species such as *Allium sativum*, *Artemisia absinthium*, and *Urtica dioica* have shown resilience and continuity in local cultural practices, maintaining medicinal, culinary, and ritual significance across the four decades. The 1983 survey documented the greatest variety of species (101), a number that decreased in subsequent studies. The 2009 survey identified 36 species not previously recorded in 1983, and the 2024 field study noted an additional 20 species. The study highlights the economic potential of several wild species in these alpine areas, such as *Achillea*, *Artemisia*, *Verbascum*, *Veronica*, *Viola*, *Polygonum*, *Bunium*, and *Sorbus* spp., which could be utilized for creating new herbal teas, artisanal beers, liqueurs, ice creams, sweets, and seasoned food products. Expanding the uses of these plants could not only preserve ethnobotanical knowledge but also stimulate local economies and support sustainable development in alpine communities. The documented temporal shifts in plant usage reflect broader cultural, ecological, and socio-economic changes, underscoring the importance of preserving biodiversity and traditional knowledge amidst ongoing environmental and societal shifts. This study underlines the need to conserve ethnobotanical heritage while adapting to the evolving landscape of the region. Future research could focus on exploring the role of these species in broader sustainability initiatives, including conservation strategies, ecosystem services, and community-based tourism while continuing to document the cultural dynamics influencing plant usage.

## 1. Introduction

The Alps are one of Europe’s most intriguing yet underexplored regions, particularly regarding the study of both tangible and intangible cultural heritage associated with traditional knowledge (TK) of plants. Despite the potential significance of such research for sustainability initiatives ranging from organic farming and home gardening to local food production, eco-tourism, eco-gastronomy, and eco-museology the impact of climate change on Alpine biodiversity and related ethnobotanical resources remains a major concern [1,2].

Traditional ecological knowledge (TEK) is vital for local communities and their well-being. TEK has been a focal point of research within the ethnobiological field, and recent developments have witnessed a shift towards a diachronic approach that emphasizes the historical evolution of these corpora of knowledge [3]. Traditional knowledge about wild plants has long served as a crucial component of cultural identity and subsistence in rural communities [4]. In mountainous regions, where ecological variability demands adaptive resilience, this knowledge is especially valuable, guiding practices related to medicine, food, and agriculture [5,6,7]. In recent years, few ethnobotanical studies in the western Alps have focused on the interactions between plant resources and human societies [8,9,10]. Within the Occitan-speaking valleys of the western Alps, specifically the Upper Varaita Valley in Italy and the Ubaye Valley in France, centuries of shared linguistic and cultural heritage have preserved a rich repository of ethnobotanical knowledge [3,11]. This knowledge is shaped not only by the region’s ecology but also by generations of social, linguistic, and cultural continuity that have persisted despite political borders and modernizing influences. While several ethnobotanical inventories have been compiled over the past five decades in various Occitan [11,12,13], Franco-Provencal, and Walser Alpine Valleys in Piedmont and surrounding areas as such Signorini and Fumagalli in 1983 [14], no field-based ethnobotanical studies have been conducted in the Varaita Valley except one study was conducted by Pieroni and Giusti (2009) [3]. Only a limited number of linguistic and ethnographic studies have been conducted in the region.

The continuity of traditional plant use within the Occitan Valleys, however, has faced new challenges in recent decades. Social, economic, and environmental changes such as depopulation, shifts in livelihood strategies, and climate variability pose risks to the intergenerational transmission of plant knowledge [15]. These factors raise questions about the resilience of traditional knowledge systems in the face of contemporary pressures, as well as about potential transformations in how local communities engage with their surrounding landscapes [16]. Documenting and analyzing the evolution of these practices offers valuable insights into the processes that influence both the persistence and adaptation of traditional knowledge.

This study focuses on the comparative ethnobotany of the Upper Varaita Valley (Bellino) in Italy and the Ubaye Valley in France. Despite the political geographic separation, these valleys share an Occitan identity that is reflected in language, folklore, and traditional practices, especially those related to plant use. Previous studies have documented aspects of this plant knowledge [3,11,14], but the current research expands on these efforts by incorporating longitudinal comparisons over four decades. By drawing on historical data, interviews, and field observations, we aim to capture shifts in plant usage and assess the extent to which traditional knowledge in these valleys has been conserved or modified over time.

The study aims to track shifts in plant usage, species diversity, and the changing roles of plants in local traditions in the western alpine mountain ecosystems. The research also seeks to understand how local communities adapt their use of wild plants in response to ecological shifts, cultural trends, and modern influences.

## 2. Results

### 2.1. Wild Plants Across the Western Alps Mountain Regions

The ethnobotanical survey of wild plants across the western Alps mountain regions reveals a rich diversity of species used for medicinal and food purposes. Table 1 presents valuable information for understanding the medicinal, food, and other uses of these plants, particularly about traditional practices in the areas being studied. For example, *Achillea erba-rotta* is used for medicinal purposes, as is *Artemisia absinthium*, *Arnica montana*, and *Gentiana acaulis*, which all show broad medicinal applications. Many plants, such as *Allium sativum* and *Mentha spicata*, are both food and medicinal, highlighting their dual-use value. The widespread use of species like *Fragaria vesca*, *Rubus idaeus*, and *Vaccinium myrtillus* for their role in local diets, particularly for their fruits, also underlines the cultural importance of wild berries in these regions (Table 1). Additionally, the occurrence of certain plants like *Artemisia genipi* and *Cetraria islandica* suggests a ritual or specialized medicinal role, further reflecting the local traditions and health practices. Notably, the field studies date back to varying periods, with some species being noted in recent studies such as the 2024 study on *Achillea erba-rotta*, while others have been documented since earlier works, such as *Achillea millefolium*, which appears in research from 2009 [3]. The transmission of plant knowledge across generations is also evident in the use of traditional names for plants, such as *Boletus edulis* and *Brassica oleracea*, which continue to be recognized for their food applications, linking past practices with contemporary uses [3,10].

The significant variations in the number of plant species used for teas across different studies and years. In [11] Novaretti and Lemordant study in 1990, 136 species were recorded, showing a broad diversity of plants utilized. Pieroni and Giusti study (2009) [3] documented 88 species, reflecting a slightly reduced diversity compared to Novaretti and Lemordant (1990) [11]. In contrast, the 2024 study identified only 66 species, indicating a noticeable decline in the diversity of utilized plants over the years.

### 2.2. Use of Wild Plants Across Study Sites in the Western Alps

**Figure 1:**
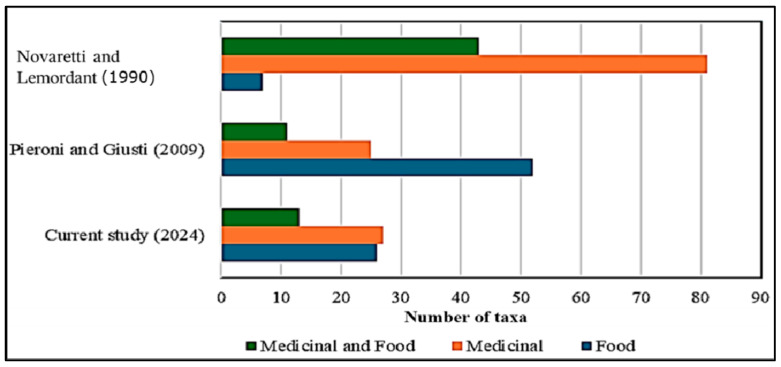
Comparative use of herbal teas for food and medicinal purposes across studies in the western Alps; Novaretti and Lemordant (1990) [11], Pieroni and Giusti (2009) [3].

The ethnobotanical survey of wild plants in the western Alps, specifically in the Ubaye and Bellino Valleys, reveals differing patterns of plant use over time (Figure 1). In the current study (2024), 26 species were primarily used for food purposes, 27 for medicinal purposes, and 13 for both medicinal and food uses. This indicates a fairly balanced use of plants for both medicinal and food purposes in present-day practices. In comparison, the study by [3] Pieroni and Giusti (2009) found a greater emphasis on food uses, with 52 species used for food purposes, 25 for medicinal purposes, and 11 for both. The earlier study by [11] Novaretti’s study shows a more pronounced reliance on medicinal plants, with 81 species used primarily for medicinal purposes, 43 for both medicinal and food purposes, and only 7 species used solely for food purposes. These findings suggest a shift in the used plants, from a primarily medicinal focus in the past to a more balanced or diversified use in the contemporary context of the western Alps. This trend reflects the dynamic relationship between the local populations of the Ubaye and Bellino Valleys and their environment, with the ongoing adaptation of traditional plant knowledge.

### 2.3. Main Ailment Categories Treated by Medicinal Plants in Western Alps

Table 2 reports the medicinal plant species comparison across three different studies focused on plant use in the western Alps, specifically the Bellino and Ubaye Valleys. The comparison includes the present study, as well as two earlier studies: Pieroni and Giusti (2009) and Novaretti’s (1990) studies [3,11]. The plants are categorized based on the classifications of [17], and the number of species reported in each study is compared.

For digestive system disorders, the present study reports 16 species, compared to 27 in Pieroni and Giusti (2009) [3] and 38 in Novaretti’s study. Common species cited in all studies include *Taraxacum campylodes*, *Allium sativum*, and *Gentiana acaulis*, which are well-known for their digestive benefits. The respiratory system disorder category shows similar species counts across studies, with 15 species reported in the present study, 16 in Pieroni and Giusti (2009) [3], and 37 in Novaretti and Lemordant study. *Mentha spicata* and *Cetraria islandica* emerge as the most frequently reported species in both the present study and Pieroni and Giusti (2009) [3]. When it comes to nutritional disorders, the present study reports 21 species, significantly more than the 16 species reported by Pieroni and Giusti (2009) [3] and 12 by Novaretti and Lemordant (1990) [11]. Plants like *Allium sativum*, *Brassica oleracea*, and *Fragaria vesca* are among the most reported for nutritional benefits. In contrast, the category for musculoskeletal system disorders shows a dramatic decrease in the number of species used in the present study, with only 1 species reported compared to 12 in the Pieroni study and 7 in the Novaretti study. The plants *Arnica montana* and *Cirsium acaule* are commonly used for musculoskeletal issues.

In the pain category, the present study reports only 2 species, while both Pieroni and Giusti (2009) [3] and Novaretti and Lemordant (1990) [11] report 9 species each. Common species used for pain relief across all studies include *Achillea millefolium* and *Artemisia indica*. The skin and subcutaneous cellular tissue disorders category also sees a reduction in the number of species reported in the present study (7 species), compared to 14 species in Pieroni and Giusti (2009) [3] and 27 species in Novaretti and Lemordant (1990) [11]. Plants such as *Arnica montana* and *Urtica dioica* are frequently mentioned for skin-related ailments.

For genitourinary system disorders, the present study reports 6 species, which is higher than the 3 species reported in Pieroni and Giusti (2009) [3] and 4 species in Novaretti and Lemordant study [11]. The species *Cynodon dactylon*, *Equisetum arvense*, and *Rumex alpinus* are the most commonly reported. Inflammation-related plant use is reported by 5 species in the present study, significantly fewer than the 26 species in Pieroni and Giusti (2009) [3] and 50 in the Novaretti and Lemordant study. *Urtica dioica*, *Artemisia absinthium*, and *Rumex obtusifolius* are among the most frequently cited species for inflammation.

For nervous system disorders, the present study reports 2 species, *Nepeta cataria* and *Valeriana officinalis*, while Pieroni and Giusti (2009) [3] and Novaretti and Lemordant [11] report 5 and 12 species, respectively. Finally, in the category of circulatory and blood system disorders, only 1 species, *Allium sativum*, is reported in the present study. In contrast, 13 species are reported in Pieroni and Giusti (2009) [3] and 18 in Novaretti and Lemordant [11], with *Juniperus communis* and *Achillea millefolium* being frequently cited in earlier studies.

These findings suggest notable shifts in the range of species used for medicinal purposes in the region over time. While some species, like *Allium sativum*, *Arnica montana*, and *Urtica dioica*, are consistently reported across all studies, the number and variety of species used for certain ailments have fluctuated. These changes may reflect shifts in ecological conditions, evolving cultural practices, or the changing availability of specific plant species in the region over time. The differences between the Bellino and Ubaye Valleys, as well as the variations between the different study periods, highlight the dynamic nature of local plant use and its adaptation over time.

### 2.4. Shifts in Traditional Plant Knowledge and Biodiversity

**Figure 2:**
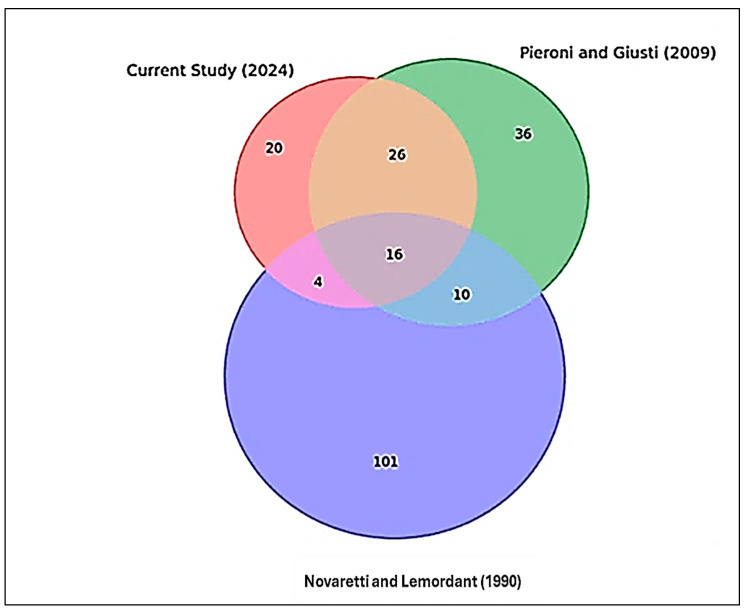
Genera overlapping within our present study and previous studies conducted in the region; Novaretti and Lemordant (1990) [11], Pieroni and Giusti (2009) [3].

The findings from the studies conducted in the Ubaye and Bellino Valleys reveal both continuity and change in the use of wild plant species. The Novaretti and Lemordant study conducted in 1983 and published in 1990 recorded the highest number of species not recorded in the recent studies, particularly in the Ubaye Valley, which accounted for 101 species and represents 47.42% of the total species found across both valleys in all three studies. The 2009 Pieroni study, which focused on the Bellino Valley, identified 36 species not recorded in the previous study, representing 16.9% of the total species recorded in both valleys. The 2024 study, however, recorded 20 species in the region, providing an updated snapshot of the ethnobotanical landscape. For more information on the scientific names and the specific localities of their presence, refer to Figure 2.

When comparing the 1983 data (published in 1990) with our current 2024 data, we found four species common to both datasets: *Equisetum arvense*, *Malva sylvestris*, *Thymus serpyllum*, and *Viola tricolor*. These plants were identified as rare in 1983 [11] and have continued to be recorded in subsequent research (Figure 2).

Sixteen plant species were common across all three studies in 1983 (conducted in 1983 but published in 1990), 2009, and 2024. Additionally, 26 species were shared between the 2024 and 2009 studies, while 10 species were common between 2009 and 1983 (published in 1990). A significant decline in species was observed between the recent and 1983 (published in 1990) studies (see Figure 2).

While the ethnobotanical traditions in these valleys remain rich, the changing patterns of plant species usage point to both the persistence of certain practices and the vulnerability of others to ecological and cultural shifts. The decline in plant biodiversity use may be linked to the decline in pastoralist activities that had maintained for centuries the exposure of locals to mountain environments, which could contribute to the diminished availability of these plants. Furthermore, as traditional knowledge about these plants becomes less widespread, the likelihood of them being passed down and utilized decreases, even if they are still present in the environment. Thus, while the species have remained part of the botanical landscape, they are increasingly “invisible” to locals and maybe even rare—possibly due also to climate and landscape changes; therefore, their declining use may be due to both environmental and cultural factors.

**Figure 3:**
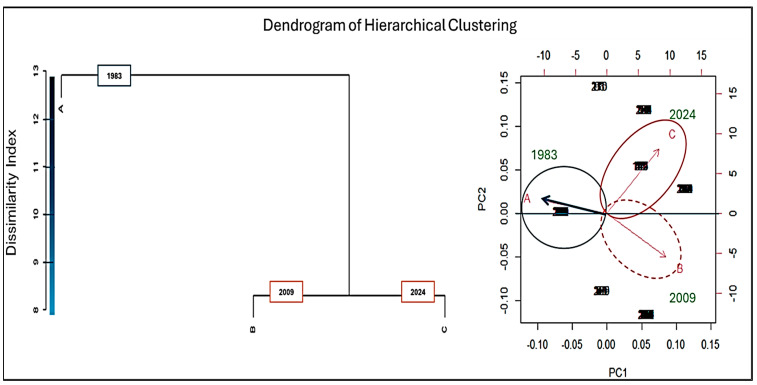
PCA biplot and clustering analysis of wild plant species used for food and medicinal purposes in the Ubaye and Bellino Valleys across three temporal periods (1983 [11], 2009 [3], and the present study in 2024). Points represent individual plant species, colored and labeled by the period of data collection. Arrows (A, B, C) denote the direction of temporal shifts in plant usage patterns, illustrating the evolution of ethnobotanical knowledge over time.

Based on the PCA and clustering analysis for the wild plant species used for food and medicinal purposes (Figure 3), we observed distinct patterns in species distribution across three temporal points: 1983 (published in 1990) (A), 2009 (B), and 2024 (C). At the ecological level, the clusters represent different combinations of key variables, including elevation, habitat type, climatic conditions, and cultural preferences for plant use. Cluster A (1983) is dominated by high-altitude species commonly found in glacial and alpine meadows. These plants were primarily used for medicinal purposes, reflecting a traditional reliance on local flora for remedies in response to limited access to pharmaceuticals. Cluster B (2009) includes species from lower altitudes and more diverse habitats, such as mixed forests and transitional zones. This reflects an expansion in plant utilization, potentially driven by changing land use practices and the increased integration of traditional and modern knowledge systems. Cluster C (2024) features species adapted to both high-altitude and valley environments. This cluster indicates a broader spectrum of usage, incorporating culinary and supplementary medicinal purposes. The expansion into cultivated and managed landscapes suggests adaptation to evolving ecological conditions and cultural preferences.

The PCA biplot shows the primary directions of variation among these points on the PC1 and PC2 axes, which capture a sizable portion of the variance in species data. Each cluster on the biplot represents groups of plant species that share similar usage and ecological distribution patterns at each of these temporal points.

In the plot, time points A, B, and C are represented as vectors, which indicate the directional changes in species distribution over time. The clusters are labeled and colored based on temporal alignment, reflecting the shifts in wild plant utilization from 1983 (published in 1990) to 2024. The dendrogram further illustrates the progression of these changes, with A (1983) as the baseline cluster, from which B (2009) and C (2024) diverge.

These findings suggest a gradual evolution in the utilization of wild plants, influenced by ecological changes, such as shifts in vegetation zones due to climate change, and socio-cultural dynamics, including changes in traditional knowledge and economic activities.

## 3. Discussion

### 3.1. Resilience and Change Wild Plants in the Western Alps

This ethnobotanical survey of wild plants in the Occitan region of the western Alps provides insight into the dynamic interplay between social and geographic factors, traditional knowledge, and the evolving cultural practices in mountain communities. Comparisons between studies conducted in the Ubaye and Bellino areas in 1983 (published in 1990), 2009, and 2024) reveal notable trends in plant usage, species resilience, and shifts in ethnobotanical practices over time.

In examining the persistence of certain plant species in the Occitan region of the western Alps, we see a clear interplay between ecological resilience, cultural significance, and the ongoing transmission of traditional knowledge. Species such as *Allium sativum*, *Artemisia absinthium*, *Brassica oleracea*, and *Sambucus nigra* have demonstrated remarkable resilience over the decades, consistently serving both medicinal and food roles. These plants have managed to withstand the challenges of the harsh alpine climate due to their ecological traits (e.g., resistance to cold and high altitudes) and the relatively low human disturbance in the region, which aids in their survival. These plants are ecologically robust and culturally integral, continuing to fulfill essential functions within local communities. Their persistence can be attributed to several factors, particularly their ecological traits and the undisturbed nature of the alpine environment.

From an ecological perspective, these species exhibit traits that enable them to thrive in the harsh mountain climate. For example, *Allium sativum* and *Artemisia indica* are well-known for their ability to withstand cold temperatures and high altitudes, while *Sambucus nigra* thrives in specific soil conditions found in the region [11,18]. These plants are adapted to endure the environmental challenges of the region, from the cold mountain winters to the relatively short growing seasons, ensuring their survival and continued presence in the landscape. Moreover, the relatively low levels of human disturbance in alpine ecosystems help preserve these species, allowing them to continue growing in their natural habitats with minimal competition from invasive species.

Culturally, these plants have maintained their importance through the transmission of traditional knowledge across generations. Plants like *Urtica dioica* and *Sambucus nigra* have long been integral to local healing practices, with their medicinal properties remaining valued by local populations. *Urtica dioica*, for instance, is known for its anti-inflammatory and detoxifying properties, while *Sambucus nigra* is frequently used in remedies for colds and respiratory issues [19,20]. The enduring relevance of these plants reflects the deeply rooted cultural knowledge that has been passed down within communities, enabling them to remain central in local practices. This cultural resilience is vital to the continued use and appreciation of these plants in the face of modern challenges.

However, despite their resilience, socio-economic changes, including tourism and the increased availability of modern medicines, have altered how these plants are used. For instance, *Artemisia absinthium*, traditionally valued for its medicinal properties in treating digestive disorders and as an antimicrobial, has seen reduced use in some areas due to the availability of pharmaceutical alternatives such as synthetic antibiotics and antacids [5,18]. Similarly, *Lavandula angustifolia*, widely used for its calming and antiseptic properties, now faces competition from synthetic calming agents and over-the-counter medications. While these plants continue to be valued, their use may now be more selective, with some species being less commonly employed due to changing preferences or the availability of synthetic substitutes.

Many of these wild plants traditionally grew in areas shaped by pastoral activities, such as pastures and meadows. These ecosystems, maintained by the grazing of livestock, created conditions that favored the growth of certain plant species [3,8]. Pastoralism, with its reliance on seasonal grazing and careful land management, promoted biodiversity by maintaining open spaces and preventing the encroachment of woody vegetation. These habitats, in turn, supported the growth of medicinal and food plants that were central to local traditions [8].

However, the decline in pastoralism in the region over recent decades has led to significant ecological changes. The reduction in grazing pressure, coupled with changing agricultural practices, has led to the overgrowth of woody plants and a decrease in open meadow habitats [21,22]. This ecological shift has affected the distribution of plant species traditionally associated with pastoral landscapes. In areas where pastoralism has decreased or been replaced by other forms of land use, such as intensive farming or forestry, the habitats that once supported these plants have been lost or altered, leading to a decline in their availability and, consequently, the erosion of local knowledge about their uses [23].

The decline in species diversity observed across the three studies from 136 species in 1983 (published in 1990) to just 66 in 2024 reflects broader ecological changes impacting the Occitan Valleys. Factors such as habitat destruction, climate change, and intensified grazing pressures in regions like Bellino have influenced the availability of plant species, consequently affecting the traditional knowledge of these plants [24,25]. For instance, species like *Petasites hybridus* have proliferated in the uncultivated areas along the Varaita stream banks, thriving in disturbed habitats and outcompeting other plants. This trend highlights how changes in land use, land management practices, and ecological conditions can reshape plant communities, leading to shifts in the diversity of ethnobotanical knowledge [12,26]. The reduction in species diversity may indicate the loss of traditional uses for certain plants, while the dominance of a few resilient species underscores the adaptability of both ecosystems and local cultural practices. In a similar study conducted by [8] by comparing wild plant knowledge in Susa Valley between 1970 and 2018, the authors found a significant decrease in wild plant knowledge, which was attributed to socioeconomic, cultural, and possibly environmental changes that occurred during the half-century.

The erosion of traditional knowledge is not solely a result of ecological changes but also reflects broader socio-economic shifts. As pastoralism has declined, younger generations have moved away from rural areas, and there has been a reduced reliance on plant-based remedies in favor of modern pharmaceuticals. This shift has contributed to the fading of traditional practices that once ensured the survival and transmission of knowledge about these plants. In some cases, younger generations may no longer engage in the collection or use of these plants, leading to the gradual loss of important ethnobotanical knowledge [3,27,28].

In contrast, the 2024 survey recorded 20 species such as *Allium sphaerocephalon*, *Amelanchier stolonifera*, *Betula pendula*, *Bistorta* spp., *Boletus edulis*, *Boletus pinophilus*, *Corylus avellana*, *Gentiana punctata*, *Lupinus albus*, *Polypodium vulgare*, *Primula vulgaris*, *Prunus avium*, and *Rumex obtusifolius*, which were not noted in previous studies. This shift in plant usage reflects the dynamic nature of local ethnobotanical practices, suggesting that while certain traditional plants continue to be used, other species have gained prominence due to numerous factors. One significant factor driving this change could be environmental alterations. Shifts in climate or land use may have led to an increase in the availability of these species, making them more accessible and, consequently, more commonly used. For example, the appearance of species like *Betula pendula* and *Boletus edulis* might be linked to specific ecological changes, such as changes in forest composition or soil conditions that favor the growth of these plants [29].

Another explanation for the rise of these species could be the resurgence of interest in natural remedies, fueled by global health trends. Modern consumers are increasingly turning to natural or plant-based solutions for health and wellness, which could lead to the renewed use of plants like *Gentiana* spp., known for their digestive benefits, or *Prunus avium*, which has a history of use in traditional medicine. The increasing demand for plant-based health products might drive the re-emergence of species that were historically used but had fallen out of favor [30].

The rise of these newly reported species highlights the adaptability of traditional plant knowledge, showing that it is not static but evolves in response to changing ecological conditions, shifting cultural practices, and evolving health needs. This adaptability ensures that traditional ethnobotanical knowledge remains relevant and continues to be passed down through generations, albeit with modifications to suit the contemporary environment. It also emphasizes that the transmission of plant knowledge is not solely about preserving past practices but also about responding to current realities and new understandings of plant-based health [18,31].

The studies document a clear shift from a predominantly medicinal use of wild plants in earlier years to a more balanced application for both food and medicinal purposes in recent times. For example, the 1983 (published in 1990) survey in Ubaye revealed that wild plants were primarily used for medicinal purposes, whereas the 2024 study in Bellino shows an almost equal distribution between food and medicinal uses. This change may be attributed to several factors, including shifts in household economy and agro-pastoral activities, urbanization, the increased availability of commercially produced medicine and also herbal remedies, dietary habits, and a growing interest in the food potential of wild plants [32,33].

### 3.2. The Economic Potential and Cultural Shifts in the Use of Wild Plants from the Western Alps

The results of this study indicate that several wild plant genera from the western Alps, including *Achillea*, *Artemisia*, *Verbascum*, *Veronica*, *Viola*, *Polygonum*, *Bunium*, *Sorbus* spp. and the quoted fungal spp. represent a promising avenue for the development of new food and beverage products. These species are widely available in the western Alps, and they are not yet fully explored in local specialty foods and beverages that make them ideal candidates for possible future artisanal productions. These new applications could not only demonstrate resilience and continuity in local cultural plant-centered practices but also possess a significant untapped potential for small-scale circular economies [34]. As the usage of these plants shifts toward culinary and medicinal purposes, there is an opportunity to expand their application into various product markets, ranging from herbal teas and tonic water to artisanal beers, liqueurs, ice creams, sweets, and seasoned food products [8]. The use of these species in creating new consumer goods could support the diversification of local economies, especially in rural and alpine communities where traditional farming practices have declined. The establishment of local artisanal entrepreneurships based on these plants could promote sustainable agriculture, preserve ethnobotanical knowledge, and create new sources of income [3,18,35]. Such products could also appeal to a growing market for natural, locally sourced, and sustainable food and beverage items, which are increasingly sought after by environmentally conscious consumers. For example, herbal teas made from *Artemisia*, *Achillea*, *Verbascum*, *Veronica*, and *Viola* spp. could tap into the wellness and functional beverage market, while liqueurs and artisanal beers aromatized by *Artemisia absinthium* and wild fruits could cater to the craft alcohol segment [36,37], and *Bunium* and *Polygonum* spp. into the wild food gastronomy.

Many traditional practices, especially those related to wild food sources, have faded with the depopulation of mountain regions. Elders, though often unaware of their role as knowledge bearers, were surprised at the interest shown by young researchers, revealing a complex relationship between past and present cultural values. Younger generations, while showing a renewed interest in ethnobotanical practices, face challenges in accessing this knowledge due to the departure of older residents and the influx of modern conveniences. The work of Giovanni Bernard in the 1986s [38], documenting interviews with the last inhabitants of Bellino’s hamlets, provides a striking comparison to the current data. His records show a stronger adherence to unique traditional uses that are now partially lost, particularly among native speakers, who have retained a more vivid connection to their origins. Post-war societal shifts saw a desire to appear modern, which contributed to the abandonment of certain traditions, including the use of wild plants for sustenance. This loss of knowledge is amplified by the migration of rural populations to urban areas, resulting in a gradual erosion of ethnobotanical traditions.

### 3.3. Limitations of the Study

The geographic scope is limited to specific valleys in the western Alps, which may not fully represent the broader region’s ethnobotanical diversity. The temporal gaps between surveys in 1983 (published in 1990), 2009, and 2024 may also affect the accuracy of the data, as some plant uses may have been lost or not recorded over time, due to possible slightly diverse methodological frames. Moreover, the reliance on “remembered” home plant remedies during interviews introduces subjectivity, and sampling bias may have resulted in underrepresenting certain demographic groups. Additionally, the study does not comprehensively address ecological changes, such as climate impacts or land-use practices, which may influence plant populations and availability. Future research should aim for broader geographic coverage, continuous data collection, and a more integrated ecological approach.

## 4. Materials and Methods

### 4.1. Study Sites and Geographical Context

**Figure 4:**
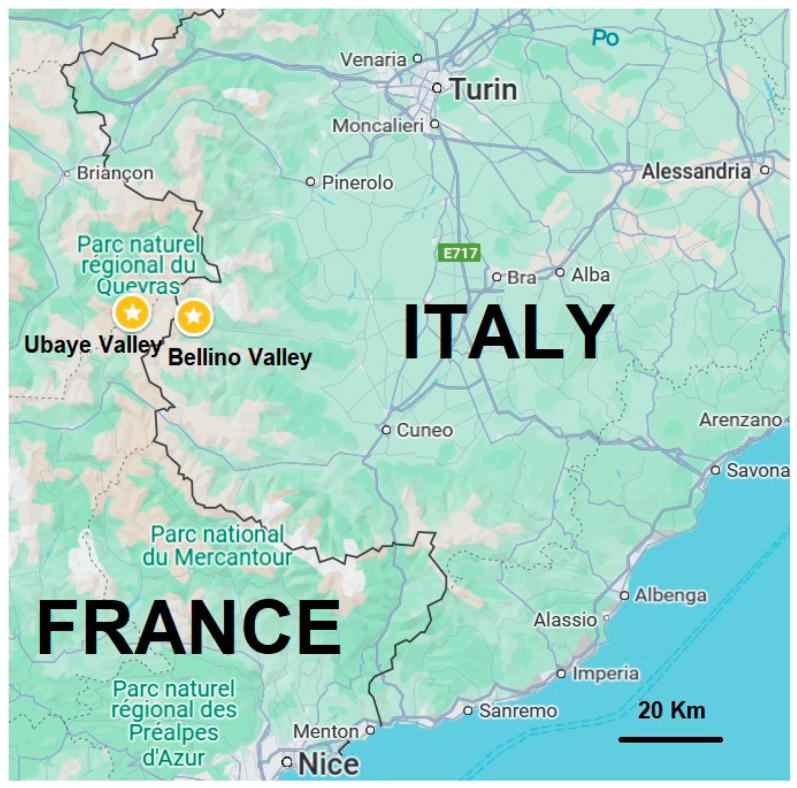
Location of the study areas (Ubaye and Bellino Valleys).

In this study, we focus on two regions with a shared Occitan linguistic and cultural heritage: Bellino Valley in Italy and Ubaye Valley in France (Figure 4). These areas, though geographically separated, share cultural and linguistic roots that provide a unique framework for comparing traditional plant knowledge.

#### 4.1.1. Bellino Valley (Italian Side)

The Bellino Valley is part of the Varaita Valley in the Cottian Alps of northwestern Italy, within the Piedmont region. It covers an area extending from Ribiera (approximately 1400 masl) to Mongioia (approximately 3340 masl) (Figure 4). This range encompasses diverse ecological zones, including glacial landscapes, sun-exposed pastures (locally known as “adrèch”), and shaded larch and Swiss pine forests (“ubàc”) [39,40]. The valley has a climate characterized by cool summers, harsh winters, and sudden fog formations locally known as “nebio stencio”, with summer temperatures averaging up to 15 °C and winter temperatures dropping as low as −10 °C, fostering a diverse range of alpine vegetation adapted to these conditions.

**Figure 5:**
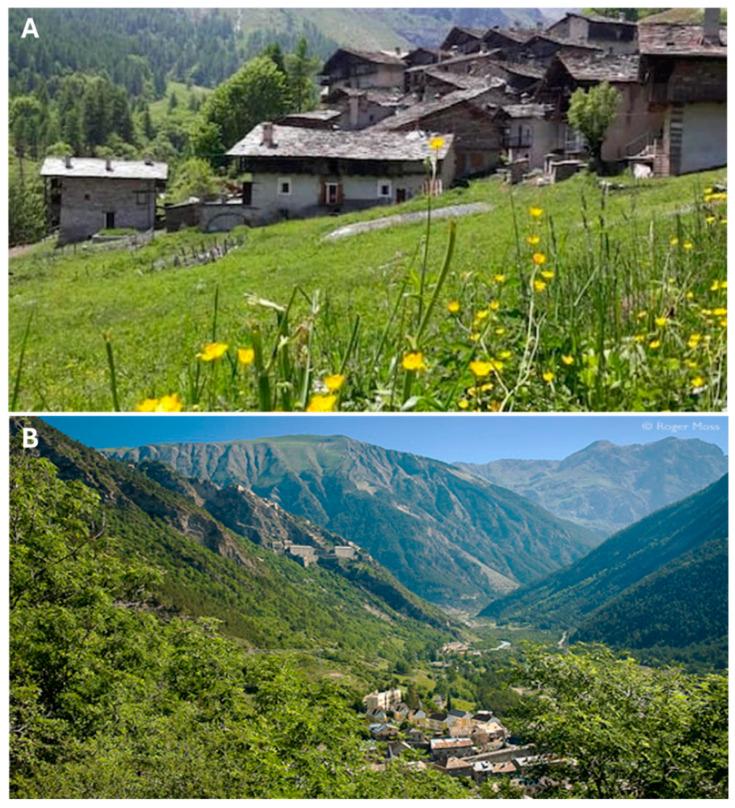
Landscape images of Bellino (**A**) and Ubaye (**B**) Valleys, showcasing their natural features, vegetation cover, and ecological diversity (photo free: Ubaye tourism site, and Wikipedia).

With a population of 107 residents that historically spoke Occitan, this region has a strong tradition of using wild plants in both food and medicinal practices. The region experiences steep gradients transitioning from densely forested areas at lower elevations to alpine meadows and bare rock zones at higher altitudes. The average temperatures range from a maximum of approximately 15 °C in summer to a minimum of −10 °C in winter. About 10% of the area is inhabited, while the remaining land is distributed as follows: 50% forest and bushes, 30% pasture, 8% cultivated area, and 2% buildings and infrastructure. These features reflect the region’s historical reliance on pastoralism, small-scale agriculture, and the gathering of wild plants for medicinal and food purposes, practices that remain vital today [3] (Figure 5A).

#### 4.1.2. Ubaye Valley (French Side)

On the French side, the study region in the Ubaye Valley in the Alpes de Haute Provence occupies an area of approximately 450 square kilometers (Figure 4), characterized by its unique geological profile of sedimentary calcareous rocks, crystalline rocks, and quartzites [11]. The valley elevation ranges from 900 to 3000 masl, with diverse landscapes that include alpine meadows, forests, and rocky terrains. The climate is alpine, with long, snowy winters and brief, mild summers, creating harsh conditions for vegetation and limiting the growing season. Average summer temperatures reach a maximum of about 15 °C, while winter temperatures can drop to as low as −10 °C. These climatic conditions, combined with the valley’s varied ecosystems, support a rich biodiversity and contribute to preserving traditional plant knowledge and ethnobotanical practices.

The valley has a population of approximately 7700 residents and retains strong cultural and linguistic ties to its Occitan heritage. Historically, the inhabitants have relied on livestock farming, high-altitude agriculture, and plant gathering for food and medicinal purposes, practices that continue to play an important role today. About 40% of the valley is covered by forests, including aspen, beech, and larch forests, especially at lower elevations. Around 35% of the area is dedicated to pasture and alpine meadows, which support grazing livestock and a variety of wild plant species. Cultivated areas make up approximately 10% of the region, with small-scale agriculture being practiced by local residents. Built-up areas, including villages and infrastructure, occupy about 5% of the valley, while the remaining 10% consists of rocky terrains, cliffs, and other natural features (Figure 5B). The climate in the Ubaye Valley is alpine, with long, snowy winters and brief, mild summers. Significant natural features, such as the Serre-Ponçon dam, the Seyne-les-Alpes Valley, and the popular tourist site of Col-Bas mark the landscape.

#### 4.1.3. Data Collection and Participant Selection

To gather comprehensive data on the ethnobotanical practices of the Bellino and Ubaye Valleys, this study used a blend of qualitative methods aimed at capturing both the current and historical dimensions of plant knowledge. Data were collected through semi-structured interviews with 30 participants in Bellino (10 men, 20 women; aged 30–85) (Table S1; Supplementary Materials). This sample was composed of older residents (65–85 years), restaurateurs (30–50 years), botanical enthusiasts, contributing to species, and compared with findings from previous studies involving 67 informants in the Varaita Valley (Italy) and 63 participants in the Ubaye Valley (France, aged 30–65). Participants were selected using purposive and snowball sampling, prioritizing culturally knowledgeable individuals.

For the Ubaye Vally a study conducted among 63 residents of the Ubaye region, many residents maintain a supply of “simples”, or medicinal plants, which they collect from both mountain pastures and valley fields. Some individuals still cultivate a variety of plants in their gardens, including *Angelica archangelica* L., *Impatiens balsamina* L., *Matricaria chamomilla* L., *Brassica oleracea*, *Anthriscus cerefolium* Hoffm., *Ribes nigrum*, *Fragaria vesca*, *Althaea officinalis*, *Artemisia genipi*, *Levisticum officinale* W.D.J.Koch, *Lilium candidum*, *Mentha spicata*, *Glycyrrhiza glabra*, *Salvia officinalis*, and *Tanacetum vulgare* [11]. On the French side, an extensive botanical survey in the Ubaye Valley was conducted. This work combined historical texts from the 18th to 20th centuries with field observations, cataloging plant species in different ecological zones.

Ethical practices were adhered to throughout the data collection process following the Code of Ethics of the International Society of Ethnobiology [41]. All participants gave verbal consent to take part in the interviews, in line with ethnobotanical research standards. This included informing participants about the purpose of the study, the confidentiality of their responses, and their right to withdraw at any time. This approach respects local customs and aligns with ethical norms for conducting fieldwork within traditional communities.

### 4.2. Data Analysis

The data used in the survey includes details on plant species, their ecological habitats, and how their usage has evolved. In terms of ecological distribution, we integrated information about each plant species’ habitat and environmental factors, drawing from interview responses and publicly available ecological data. Non-parametric observations, such as qualitative habitat descriptions and usage patterns (e.g., “frequently used”, “occasionally used”), were systematically converted into parametric variables.

Categorical responses were assigned numerical scores, allowing us to quantify trends in plant usage. Habitat descriptions were linked to corresponding environmental parameters, such as elevation, and precipitation levels, sourced from ecological datasets. These transformations enabled us to identify the ecological zones where each plant species is commonly found. To ensure comparability across variables, ecological and usage data were standardized using z-scores. For variables lacking inherent parametric distributions, smoothing techniques such as kernel density estimation (KDE) were applied to approximate continuous distributions. The transformed data were validated for normality using tests like the Shapiro-Wilk test and, where necessary, robust statistical methods such as generalized linear models were employed.

To systematically analyze the ethnobotanical data, we adopted a multifaceted approach combining quantitative, qualitative, and comparative methods to discern patterns in traditional plant use and observe historical continuity and shifts in ethnobotanical knowledge. All analyses were conducted in R (version 4.4.1) and SAS 9.4. We conducted a frequency analysis to compare the prevalence of specific plants or categories (e.g., medicinal, food) across the three datasets (1983 (published in 1990), 2009, and 2024). Chi-square and Fisher’s exact tests were applied for pairwise comparisons of plant presence/absence across periods. McNemar’s test was used to identify significant shifts in plant presence in paired datasets (identical species across all periods). The significance threshold was set at *p* < 0.05.

Cluster analysis and principal component analysis (PCA) were conducted to group plant species by region and period, helping to identify unique plant usage patterns within each valley and time period.

For qualitative analysis, interview transcripts and observation notes were systematically coded to identify recurring themes, such as medicinal applications, culinary uses, and symbolic traditions associated with specific plants. Comparative indices, such as similarity Index, were applied to evaluate the overlap and divergence in plant use between the Bellino and Ubaye Valleys.

Plants list was cross-referenced with established botanical databases, such as The World Flora Online (WFO) Plant List, to validate scientific nomenclature and authorship [42]. When possible, voucher specimens were prepared, and identification was confirmed using standard taxonomic keys.

## 5. Conclusions

This ethnobotanical survey reveals significant shifts in the traditional use of wild plants in the western Alps, with a transition from primarily medicinal to more balanced food and medicinal applications over time. Despite a decline in species diversity, certain plants have shown resilience and continue to hold cultural value. New species recorded in 2024 suggest evolving traditional practices. The study also emphasizes the economic potential of these plants for local development, with opportunities in herbal teas, artisanal beverages, and gourmet food products. Future research should explore the commercialization of these species, including value-added products like seasoned pastas and cured meats, and investigate their flavor profiles and medicinal properties. Collaboration between researchers, local communities, and entrepreneurs can foster a sustainable local economy, preserving the region’s ethnobotanical heritage.

## Figures and Tables

**Table 1:** Recorded food and medicinal plants and some fungus used in the three studies (1: Present, 0: Absent; +++ quoted by more than 40% of the study participants; ++ quoted by 10–39% of the study participants; + quoted only by 1, 2, or 3 study participants).

Plant Species	Novaretti and Lemordant (1990) [11]	Pieroni and Giusti (2009) [3]	Current Study (2024)	Local Name	Parts Used	Mode of Preparation; Medical Use	Frequency of Citation
*Abies alba* Mill.	1	0	0	Sapin, Sapino, Sap Bijoun, Épicéa, Serenta, Serento, Pega, Pegoumas, Chatarela, Coucareou, Coueasela, Pinso, Thea	Resin, bud	Used in poultices for healing and respiratory issues	++
*Achillea erba-rotta* All.	1	1	1	Canamìo, Rua	Dried flowering aerial parts	Used for its anti-inflammatory properties, treating wounds and digestive issues	+++
*Achillea millefolium* L.	1	1	1	Erbo taioouro	Flowers	Tea for colds; used in soups for flavor and healing properties	++
*Agrimonia parviflora* Aiton	1	0	0	Aigremoine, Erb de Sant-Gui	Dry or fresh leaves and flowering tops	Used for digestive and respiratory health	+
*Alchemilla vulgaris* L.	0	1	0	Gunnellettes	Dried leaves	Used to treat women’s health issues, such as menstrual problems and skin conditions	+
*Allium ampeloprasum* L.	1	1	1	Por	Bulb	Used in soups, stews, and salads for flavor	++
*Allium cepa* L.	1	1	0	Seoulo	Fresh bulbs	Commonly used in cooking to add flavor to dishes, especially soups and stews	+++
*Allium sativum* L.	1	1	1	Ai	Bulb	Garlic for immune boosting; used in cooking for its antimicrobial properties	+++
*Allium schoenoprasum* L.	0	1	1	Poureto	Leaves	Used fresh in salads, soups, and as a garnish	+++
*Allium sphaerocephalon* L.	0	0	1	Aiet	Bulb	Used in soups and stews for flavor	++
*Althaea officinalis* L.	1	0	0	Guimauve, Mauve, Guimauve officinal	Flowers, leaves, roots	Used to soothe sore throats and as a mucilage	+
*Amelanchier stolonifera* Wiegand	0	0	1	Ramenchier	Berries	Eaten raw; used in jams, jellies, and pies	++
*Anchusa azurea* Mill.	1	0	0	Bourrigai, Causidan	Dry flowers and flowery tops	Used for soothing inflammation and healing wounds	+
*Anchusa officinalis* L.	1	0	0	Anchoritotico	Dry leaves	Used for wound healing and skin irritation	+
*Angelica sylvestris* L.	1	0	0	Angélique sauvage, Cournacho	Stem	Used for digestive support and to treat nausea	++
*Antennaria dioica* (L.) Gaertn.	0	1	0	Piot de chat	Dried flowers, aerial parts	Used for its medicinal properties, particularly for women’s health issues	+
*Arctium lappa* L.	1	0	0	Bardana, Alapas, Bardarie	Dry roots	Blood purifier; detoxifying; skin issues	+
*Arctium minus* (Hill) Bernh.	1	0	0	Erbo de la Jaunisso Lampourd	Dry roots	Anti-inflammatory; skin conditions	+
*Arctium tomentosum* Mill.	1	0	0	Glouteroun	Fresh fruit	Digestive; anti-inflammatory	+
*Aria edulis* (Willd.) M.Roem.	0	1	1	Alier	Berries	Used in traditional remedies for improving digestion and treating colds	+
*Arnica montana* L.	0	1	1	Strunharelo	Flowers	Used in topical ointments for bruises, sprains, and muscle pain	+++
*Artemisia absinthium* L.	1	1	1	Erbo bioncho	Leaves	Used as a digestive aid and in tinctures for appetite stimulation	+
*Artemisia genipi* Stechm.	0	1	1	Genepì maschi	Flowers	Used in alpine rituals, as a bitter aperitif, and in herbal liqueurs	+++
*Artemisia granatensis* Boiss.	1	1	1	Genepì fumelo	Leaves	Used in teas for digestive health and as a tonic	++
*Artemisia indica* Willd.	0	1	1	Artumiso	Dried aerial parts	Used for its ability to relieve menstrual pain and improve digestion	+++
*Artemisia umbelliformis* Lam.	1	1	0	Genepì fumelo	Dried flowers, aerial parts	Used for its digestive, antispasmodic, and anti-inflammatory properties	++
*Asparagus officinalis* L.	1	0	0	Asperge, Aspergo, shoot	Roots stock, dry roots	Used as a diuretic and in savory dishes, particularly in salads and soups	+
*Astragalus glycyphyllos* L.	1	0	0	Astragalea feuille	Leaves, roots, seed	Used for immune system support and energy	+
*Avena sativa* L.	0	1	1	Aveno	Grains	Used in porridge, baked goods, and as a soothing tea for relaxation	+++
*Berberis vulgaris* L.	1	0	0	Aigret, Aigrebouet	Roots bark, ripe fruit	Used for digestive health and in jams	++
*Betula pendula* Roth.	0	0	1	Biulo	Bark	Used in teas for diuretic effects and to ease inflammation	++
*Bistorta* spp.	0	0	1	Ambouino	Roots	Tea for digestive issues and as an anti-inflammatory	+
*Bistorta bistortoides* (Pursh) Small	1	1	0	Zonbuines, Lingo boino, Linboina	Fresh young leaves	Leaves used in local salads and dishes	+++
*Boletus edulis* Bull.	0	0	1	Bulè	Mushrooms	Used in soups, sauces, and risottos for its rich, earthy flavor	+++
*Boletus pinophilus* Pilát & Dermek	0	0	1	Muscatè	Mushrooms	Used in food dishes, especially sauces and soups	++
*Boletus* spp.	0	1	0	Boulet	Fruiting body	A highly valued mushroom used in various dishes, particularly stews and soups	+
*Borago officinalis* L.	1	0	0	Bourrache, Bourrigai	Fresh flowers	Used in teas for promoting skin health and reducing fever	++
*Brassica oleracea* L.	1	1	1	Chaoul	Leaves	Used in salads, soups, and as a steamed vegetable	+++
*Bryonia alba* L.	1	0	0	Boudougno, Brionia, Briouina, Coucoumelessa	Dry roots	Diuretic; anti-inflammatory; purgative	+
*Bunium bulbocastanum* L.	0	1	1	Gravaioun	Roots	Root used in teas for digestive problems; also used in cooking for flavor	+
*Buxus sempervirens* L.	1	0	0	Buis	Whole plant	Used in traditional medicine for respiratory problems	++
*Capsella bursa-pastoris* Medik.	1	0	0	Bourse-à-Pasteur	Fresh whole plant	Used for healing wounds and digestive disorders	++
*Carlina acaulis* L.	0	1	0	Chardousso	Fresh flower receptacles	Used in traditional dishes for its aromatic properties	++
*Carum carvi* L.	0	1	1	Grane de serieis	Seeds	Used in baking (e.g., rye bread) and as a digestive aid in tea	+
*Centaurea cyanus* L.	1	0	0	Blavet, Blavetas, Bluiret, Bluget	Dry roots	Anti-inflammatory; wound healing	+
*Cetraria islandica* (L.) Ach.	1	1	1	Gratacoul	Dried thallus	Used in traditional soups, especially in Scandinavian cuisine	++
*Chamomilla recutita* var. *recutita*	1	0	0	Camomilha, Camomille	Flowery tops	Calming; anti-inflammatory; digestive	++
*Chelidonium majus* L.	1	1	0	Erbo de Santo Mario	Fresh latex	Used in traditional remedies for warts and digestive problems	+
*Chenopodium bonus-henricus* (L.) Rchb.	1	1	1	Bies	Leaves	Used in salads, soups, and as a cooked green similar to spinach	+++
*Cichorium intybus* L.	1	0	0	Chicorée, Chicourera	Flowers, leaves, roots	Digestive; appetite stimulant; liver health	++
*Cirsium acaule* (L.) A.A.Weber ex Wigg.	0	1	1	Vazanel	Leaves	Leaves used in poultices for skin irritations and as a mild diuretic	++
*Cirsium arvense* (L.) Scop	0	1	0	Choussio	Fresh young leaves	Leaves used in salads and as a pot herb	+
*Cirsium spinosissimum* Scop.	0	1	0	Chardousso	Fresh flower receptacles	Tender flowers used in cooking, similar to artichokes	++
*Corylus avellana* L.	0	0	1	Ooulanher	Nuts	Eaten raw, roasted, or used in sweets and baking	+++
*Crataegus monogyna* Jacq.	1	0	0	Aubépine, Soubevieto, Arsinat	Flowers and berries	Used for heart health and in jams	++
*Cynodon dactylon* (L.) Pers.	0	1	1	Gramigno	Leaves	Tea for urinary issues; leaves used in food dishes like salads	++
*Dryas octopetala* L.	1	0	0	The des Alpes	Flowers tops and leaves	Used for respiratory ailments and digestive issues	+++
*Echium italicum* L.	0	1	0	Erbo vieio	Fresh young leaves	Used for its anti-inflammatory and healing properties	+
*Echium vulgare* L.	1	0	0	Viperina, Viperine	Dry flowers and flowery tops	Known for respiratory benefits and treating skin conditions	+
*Elymus repens* (L.) Gould	1	0	0	Bauco-courriolo, Chiendent, Gràm, Gramé, Gramoùn	Rhizome	Used for its diuretic and anti-inflammatory properties	+
*Equisetum arvense* L.	1	0	1	Arbouret	Stems	Used in teas for urinary health, skin, and hair benefits	+
*Euphrasia officinalis* L.	1	0	0	Euphraise, Herba	Aerial parts	Used for treating eye infections and as an anti-inflammatory	+
*Fagopyrum esculentum* Moench	0	1	0	Furmetin, Pignoulet	Fruits	Used in making buckwheat flour, pancakes, and porridge	+++
*Ficus carica* L.	1	0	0	Abicou, Abicoul, Figa, I’iguier	Fruit, sap	Used for digestive health and in jams, dried fruits, and cooking	+
*Fragaria vesca* L.	1	1	1	Frole	Berries	Eaten fresh; used in jams, jellies, and desserts	+++
*Frangula alnus* Mill.	1	0	0	Bourdaine, Nerprun des Alpes	Bark, leaves	Laxative and used to treat constipation	+
*Fraxinus excelsior* L.	1	0	0	Cantharidier	Fresh Leaves or dry	Known for its anti-inflammatory and diuretic properties	++
*Fumaria officinalis* L.	1	0	0	Fumeterre	Fresh whole plant	Used for liver detoxification and digestive issues	+
*Gentiana acaulis* L.	0	1	1	Gensianelo/chaouso de cucù	Flowers	Used in digestive tonics and bitters; promotes appetite	+++
*Gentiana lutea* L.	1	1	0	Ergensano	Dried roots	Roots used in herbal liqueurs for digestive health	+++
*Gentiana punctata* L.	0	0	1	Ergensano	Flowers	Tea for digestive disorders; often used in bitters	++
*Glycyrrhiza glabra* L.	1	0	0	Réglisse, Liquirizia	Roots	Used for digestive health, in teas, and as a sweetener	+
*Gynostemma pentaphyllum* (Thunb.) Makino	1	0	0	Ginseng de l’Ouest, Herbe d’immortalité	Fresh whole plant	Known for boosting immunity and overall vitality	+
*Hedera helix* L.	1	0	0	Cuné, Curré, Lierre, Lierre grimpant	Leaves	Used for respiratory ailments, cough, and bronchitis	++
*Heracleum sphondylium* L.	0	1	0	Turmel	Fresh aerial parts	Used to treat colds, coughs, and digestive issues	+
*Herniaria glabra* L.	1	0	0	Blanqueta	Whole plant	Used for its diuretic properties	+
*Hordeum vulgare* L.	0	1	1	Uerge	Grains	Used in porridge, bread, and beer making	+++
*Hypericum perforatum* L.	1	1	0	Erbo di San Juan	Fresh flowers, aerial parts	Used for its antidepressant properties and as a wound healer	+
*Hyssopus officinalis* L.	1	0	0	Isop, Goupillon, Hysope, Marimas	Flowers tops and leaves	Used for respiratory issues, digestive health, and as a food herb	++
*Juglans regia* L.	1	1	1	Nounzal	Nuts	Eaten raw or used in baking and desserts	+++
*Juniperus communis* L.	1	1	0	Chais	Dried fruits	Berries used to flavor meat dishes and in the making of gin	++
*Juniperus oxycedrus* L.	1	0	0	Bougnet, Cade, Cade picant, Genevrier Cade, Oli de Cade	Oil	Oil used for its antiseptic and diuretic properties	++
*Laburnum alpinum* (Mill.) Bercht. & J.Presl	0	1	0	Albourn	Fresh flowers, aerial parts	Used in traditional medicine for treating respiratory issues	++
*Larix decidua* (L.) Mill.	0	1	0	Melze, Merze	Resin	Resin used in making traditional liqueurs and for flavoring	+++
*Lathyrus* spp.	0	1	0	Lioum	Seeds	Seeds used in soups and stews	+
*Lavandula angustifolia* Mill.	1	0	0	Lavande, Lavado femelo	Flowery tops	Known for calming effects, aiding sleep, and as a food herb	++
*Lilium candidum* L.	1	0	0	Lis de la Madone, Madonna Lily	Bulb, flowers	Bulb used for its soothing and anti-inflammatory properties	+
*Linum usitatissimum* L.	1	1	0	Lin	Seeds	Seeds used for oil production and in baking	+
*Lupinus albus* L.	0	0	1	Lioum	Seeds	Used in soups, salads, and as a protein source	++
*Malva neglecta* Wallr.	0	1	0	Rioundelo	Leaves	Leaves used in salads and soups, valued for their mild flavor	+++
*Malva sylvestris* L.	1	0	1	Rioundelo	Leaves	Used in teas for soothing sore throats and respiratory issues	+++
*Melissa officinalis* L.	1	0	0	Melisse, Citronnelle	Fresh or dry leaves	Known for calming nerves and aiding digestion	++
*Mentha longifolia* (L.) L.	1	0	0	Menthe douce	Leaves	Used for digestive and respiratory health	++
*Mentha spicata* L.	1	1	1	Mento	Leaves	Used in teas for digestive issues and as a flavoring in food dishes	+++
*Mentha viridis* L.	1	0	0	Menthe verte, Spearmint	Aerial parts	Digestive; refreshing; aromatic	++
*Myrrhis odorata* (L.) Scop.	0	1	0	Charvei	Aerial parts	Used in desserts and flavoring, especially in traditional European dishes	+
*Narcissus poeticus* L.	0	1	0	Susarelo, Joes de mel, Fior del mel	Flowers	Used in perfumes and for their sedative, anti-anxiety effects	+
*Nasturtium officinale* R. Br.	0	1	1	Creseintin	Leaves	Used in salads and as a garnish; also has antibacterial properties	++
*Nepeta cataria* L.	0	1	1	Erbo chato	Leaves	Used as a calming tea for anxiety and stress	++
*Onobrychis montana* DC.	0	1	0	Sparsei	Fresh and dried aerial parts	Used to relieve pain and aid in digestion	+
*Origanum vulgare* L.	1	0	0	Origan, Ourigan	Flowers and leaves	Used for respiratory health and as a food herb	++
*Papaver rhoeas* L.	1	0	0	Coquelicot	Petal and capsule	Used to soothe coughs and as a mild sedative	+
*Paronychia argentea* Lam.	1	0	0	Panado	Aerial parts	Used for treating skin ailments and wounds	+
*Persicaria hydropiper* (L.) Spach	1	0	0	Pacienco, Petite oseille	Leaves	Used for digestive and anti-inflammatory purposes, and in sauces	+
*Petasites hybridus* (L.) G.Gaertn., B.Mey. & Scherb.	0	1	1	Ciapus	Leaves	Used for migraine relief, also in teas for respiratory and skin issues	+++
*Petroselinum crispum* (Mill.) Fuss.	1	0	0	Persil, Givert, Jouveart, Juvert, Cerfeuil	Fresh leaves	Used in cooking for flavor and as a garnish	++
*Peucedanum ostruthium* (L.) W.D.J. Koch	0	1	0	Algrot	Fresh roots (reis)	Roots used in traditional mountain dishes	+++
*Picea abies* (L.) H.Karst.	1	0	0		Resin, bud		+++
*Pinus cembra* L.	0	1	1	Elvou	Needles	Used in teas for respiratory issues and as a general tonic	+++
*Pinus sylvestris* L.	1	0	0		Resin, bud		+++
*Piper nigrum* L.	0	1	0	Pepe	Dried fruits	Used for its medicinal properties, often for digestive issues	+
*Plantago lanceolata* L.	1	0	0	Maguma, Mariar d’ase, Pebre d’ay	Leaves, aerial parts	Used for treating coughs and respiratory ailments	++
*Plantago major* L.	1	0	0	Goupillon, Plantain, Pebre d’ai	Leaves, flowery tops	Used for treating wounds and inflammation	++
*Plantago media* L.	1	0	0	Isop, Lisep, Mairarinas	Leaves, flowery tops	Known for soothing respiratory issues and inflammation	+
*Polygonum aviculare* L.	1	0	0	Cigreto	Whole plant	Used for urinary tract infections and in salads.	++
*Polypodium vulgare* L.	0	0	1	Ergalisio	Leaves	Tea for lung health, used for treating coughs and chest congestion	+
*Primula auricula* L.	1	0	0	Primevere des bois, Petite primevere	Fresh flowers	Used for respiratory issues and as a mild sedative	+
*Primula veris* subsp. *macrocalyx* (Bunge) Lüdi	1	0	0	Primevere, Coueou, Jaeq, Couguou	Dry flowers	Used in treating respiratory conditions like colds and coughs	++
*Primula vulgaris* Huds.	0	0	1	Petite primevere	Aerial parts, flowers	Cough; respiratory issues; soothing	+
*Prunus avium* (L.) L.	0	0	1	Blin	Flowers	Used in teas for treating coughs and colds	+++
*Prunus domestica* L.	0	1	0	Brigne	Fresh fruits	Plums used fresh in a variety of dishes	+
*Prunus spinosa* L.	1	0	0	Prunellier, Aigras, Agrouséla	Ripe fruit	Used in jams, jellies, and drinks	++
*Pulmonaria angustifolia* L.	1	0	0	Pulmonaria tuberosa, Pulmonaria	Dry leaves	Known for treating lung conditions and as an anti-inflammatory	++
*Pulmonaria officinalis* L.	1	0	0	Pulmonaire, Herba depalmoun	Flowery summit	Used for treating coughs and respiratory disorders	++
*Rhododendron ferrugineum* L.	1	0	0	Ambruisa, Boujen, Bourenc, Bourjinquin, Lambsa	Dry leaves, fresh flowers	Used for inflammation and pain relief	+
*Ribes nigrum* L.	1	0	0	Cassis	Leaves or fruit	Used for its antioxidant properties, in jams and juices	++
*Ribes rubrum* L.	1	0	0	Groseiller rouge	Leaves or fruit	Used to treat infections and in desserts	++
*Rosa canina* L.	1	1	1	Gratocul	Fruits	Used in teas for immune support; used in jams and syrups	+++
*Rubus fruticosus* Lour.	1	0	0	Ronce, Baie sauvage	Fresh or dry leaves	Used for jams, syrups, and teas	+++
*Rubus idaeus* L.	1	1	1	Amou	Berries	Eaten fresh; used in jams, desserts, and juices	+++
*Rumex acetosa* L.	1	1	1	Setou	Leaves	Used in salads, soups, and as a digestive aid in teas	+
*Rumex acetosella* L.	1	0	0	Citarla, Grande oseille, Oseille	Leaves	Used for digestive issues, and in soups and salads for its tangy flavor	++
*Rumex alpinus* L.	0	1	1	Gravaso	Leaves	Used in teas for kidney health and as a mild laxative	++
*Rumex obtusifolius* L.	0	0	1	Rembou	Leaves	Tea for inflammation and as a detoxifier	+
*Ruta graveolens* L.	1	0	0	Ruda, Rue fétide	Flowers and leaves	Applied for digestive issues; also used for insect repellent	+
*Salvia officinalis* L.	1	0	0	Sauge, Salvia	Fresh leaves	Known for treating digestive issues and as a flavoring herb	++
*Sambucus nigra* L.	1	1	1	Sambuc	Flowers, berries	Tea for colds and flu; berries used in syrups, jams, and wine-making	+++
*Satureja montana* L.	1	0	0	Peber d ase, Sabrugra, Sabruicha	Aerial parts	Used for digestive health and as an anti-inflammatory	++
*Secale cereale* L.	0	1	1	Seil	Grains	Used in bread, porridge, and beer production	+++
*Sedum album* L.	0	1	0	Salabron	Fresh aerial parts	Used for its diuretic properties and in teas	+
*Sempervivum alatum* Scheele	1	0	0	Erboi veire, Saudo-quieú	Aerial parts	Skin care; wound healing	+
*Sempervivum montanum* L.	1	0	0	Houseleek des montagnes	Whole plant	Used for wound healing and to soothe burns	+
*Sempervivum tectorum* L.	1	0	0	Artichaut-fer, Houseleek	Fresh leaves	Used for skin and wound healing	++
*Silene vulgaris* (Moench) Garcke	0	0	1	Cuiet	Leaves	Used in soups and as a vegetable in cooking	++
*Solanum dulcamara* L.	1	0	0	Douce-amère, Dousamera, Moureletto	Dry stem	Used for skin conditions and inflammation	+
*Solanum tuberosum* L.	0	1	0	Trufes	Fresh tubers	Potatoes used in a variety of food applications	+++
*Sorbus aucuparia* L.	0	1	0	Alier	Fresh fruits	Fruits used in teas and as flavorings for jams	+
*Spergula arvensis* L.	1	0	0	Pizzera	Fresh fruits	Berries used fresh or preserved for food purposes	+
*Spiraea* spp.	1	0	0	Pousse de sel, Pousse de terre	Whole plant	Used for respiratory issues	+
*Stachys recta* L.	1	0	0	Spirae de montagne	Flowery tops	Used for digestive and anti-inflammatory benefits	++
*Suillus luteus* (L.) Roussel.	0	0	1	Ail de prairie	Aerial parts	Known for treating respiratory conditions	+
*Tanacetum parthenium* (L.) Sch.Bip.	1	0	0	Bouton d’Argent, Camamieri, Camomille	Flowers or aerial parts	Anti-inflammatory; fever reducer, headache relief	+
*Tanacetum vulgare* L.	1	1	0	Pinol	Mushrooms	Used in stews, soups, and sauces for its meaty texture and earthy flavor	++
*Taraxacum campylodes* G.E.Haglund	1	1	1	Tarasacum, Dent deliun	Leaves, flowers, roots	Detoxifying; diuretic; digestive	++
*Teucrium chamaedrys* L.	1	0	0	Calamendrié, Germandrée, Petit Chêne	Aerial part	Used for digestive support and as an anti-inflammatory	+
*Thymus pulegioides* L.	0	1	0	Serpour, Serpoul	Dried leaves	Used for its medicinal properties and flavoring in Mediterranean dishes	++
*Thymus serpyllum* L.	1	0	1	Serpoul	Leaves	Used in teas for respiratory issues and as a flavoring in food dishes	+++
*Thymus vulgaris* L.	1	0	0	Thym	Aerial parts	Used for respiratory health and as a food herb	++
*Tilia cordata* Mill.	1	0	0	Tilleul officinal	Flowers and sap	Used for calming effects in teas, and for respiratory health	+++
*Tilia platyphyllos* Scop.	1	0	0	Tilleu	Flowers and sap	Used for calming teas and respiratory benefits	++
*Tragopogon pratensis* L.	0	1	1	Barbobuc	Roots	Roots used in teas for digestive health and mild laxative properties	+
*Tripleurospermum inodorum* (L.) Sch.Bip.	1	0	0	Matricaria	Flowery tops	Anti-inflammatory; pain relief; soothing	+
*Triticum aestivum* L.	0	0	1	Bia	Grains	Used in baking (bread, cakes), porridge, and as a staple food ingredient	+++
*Tussilago farfara* L.	1	1	0	Pupettes	Dried flowers	Used for its medicinal properties, especially for respiratory issues	+
*Urtica dioica* L.	1	1	1	Urtio	Leaves	Leaves used in soups, stews, and teas for detox, anti-inflammatory benefits, and as a food ingredient	+++
*Urtica urens* L.	1	0	0	Ortie, Petite ortie	Whole plant	Used for treating joint pain, rich in iron, and used in soups and teas	++
*Vaccinium myrtillus* L.	1	1	1	Aize	Fresh fruits	Berries used in medicinal teas and desserts	+++
*Vaccinium uliginosum* L.	0	1	0	Poumarette	Fresh fruits	Berries used for making jams, teas, and syrups	++
*Vaccinium vitis-idaea* L.	0	1	1	Pet merlet	Berries	Berries used in jams, sauces, and as a flavoring in various dishes	++
*Valeriana officinalis* L.	1	0	0	Valeriana, Valeriano	Dry roots	Used for anxiety, insomnia, and stress relief	+
*Valerianella* spp.	0	1	0	Salzet	Fresh young leaves	Used in salads, often referred to as corn salad	+
*Veratrum album* L.	0	1	0	Varaire	Roots	Used for its potent medicinal properties, particularly for heart issues	+
*Verbascum densiflorum* Bertol.	0	0	1	Nevioun	Flowers	Flowers used in teas to treat respiratory issues, such as coughs and colds	++
*Verbascum lychnitis*	1	0	0	Great mullein, Yellow mullein	Flowers, leaves	Respiratory issues; expectorant; anti-inflammatory	+
*Verbascum nigrum* L.	0	1	0	Nevioun	Flowers	Flowers used for their soothing properties for respiratory issues	+
*Verbascum phlomoides* L.	1	0	0	Clergi de nostro Damo, Farieu	Dry leaves	Used for respiratory health, especially for coughs	+
*Verbascum pulverulentum* Vill.	1	0	0	Varlaco, Yezéoscutum	Dry flowers	Helps with respiratory ailments and inflammation	+
*Verbascum thapsus* L.	1	0	0	Bouillon blanc, Bouihoun blanc	Dry flowers	Known for respiratory support and treating coughs	+
*Veronica allionii* Vill.	0	1	1	Jas per tero	Leaves	Leaves used in teas for digestive health and as an anti-inflammatory	+
*Veronica beccabunga* L.	0	1	0	Seiraset	Leaves	Used in salads and traditional mountain dishes	+
*Veronica officinalis* L.	1	0	0	Véronique	Whole plant	Used for treating wounds and digestive disorders	++
*Vicia faba* L.	0	1	1	Favo	Seeds	Seeds used in soups, stews, and as a protein-rich ingredient in various dishes	++
*Vicia lens* (L.) Coss. & Germ.	0	1	0	Lentie	Seeds	Used in soups, stews, and salads	++
*Viola calcarata* L.	1	0	0	Violette des calanques	Whole plant	Used for respiratory issues and skin conditions	+
*Viola canina* L.	0	0	1		Flowers, leaves		
*Viola hirta* L.	0	0	1	Viouleto	Flowers, leaves	Leaves and flowers used in teas to treat skin ailments and respiratory issues	+++
*Viola odorata* L.	1	1	0	Vioulette	Dried leaves, leaves	Used in teas and as a food-flavoring herb	++
*Viola tricolor* L.	1	0	1	Viouleto	Flowers	Used as a tea against the Spanish flu	+++
*Viscum album* L.	1	0	0	Gui blanc	Whole plant	Used to lower blood pressure and treat varicose veins	+
*Vitis vinifera* L.	0	1	0	Vinegar	Poultice with clay	Used in poultices for wound healing and skin care	++
*Zea mays* L.	0	1	0	Meliga	Seeds	Corn used in cooking, typically in cornmeal or as a vegetable	+++
Unidentified taxon	0	1	0	Erbo dousso	Fresh aerial parts	Wild herb used in local dishes	+

**Table 2:** Aliment categories based on the respondents’ reports.

Medicinal Use Category	Number of Reported Species			Most Reported Species Across Studies
	Current Study (2024)	Pieroni and Giusti Study (2009) [3]	Novaretti Study (1990) [11]	
Digestive system disorders	16	27	38	*Allium sativum*, *Artemisia absinthium*, *Carum carvi*, *Gentiana acaulis*, *Mentha spicata*, *Taraxacum campylodes*
Respiratory system disorders	15	16	37	*Cetraria islandica*, *Juniperus communis*, *Mentha spicata*, *Plantago lanceolata*, *Sambucus nigra*, *Thymus serpyllum*, *Tussilago farfara*
Nutritional disorders	21	16	12	*Allium sativum*, *Brassica oleracea*, *Fragaria vesca*, *Hordeum vulgare*, *Linum usitatissimum*, *Prunus avium*, *Rubus idaeus*, *Secale cereale*, *Vaccinium myrtillus*
Musculoskeletal system disorders	1	12	7	*Arnica montana*, *Cirsium acaule*, *Cirsium arvense*, *Cirsium spinosissimum*, *Equisetum arvense*, *Gentiana lutea*, *Heracleum sphondylium*
Pain	2	9	9	*Achillea millefolium*, *Artemisia absinthium*, *Artemisia indica*, *Arnica montana*, *Chelidonium majus*, *Tanacetum vulgare*
Skin/subcutaneous cellular tissue disorders	7	14	27	*Achillea erba-rotta*, *Arnica montana*, *Cirsium acaule*, *Juglans regia*, *Nasturtium officinale*, *Urtica dioica*, *Viola tricolor*
Genitourinary system disorders	6	3	4	*Cynodon dactylon*, *Equisetum arvense*, *Rumex alpinus*
Inflammation	5	26	50	*Arnica montana*, *Artemisia absinthium*, *Rumex obtusifolius*, *Taraxacum campylodes*, *Urtica dioica*, *Veronica allionii*
Nervous system disorders	2	5	12	*Hypericum perforatum*, *Nepeta cataria*, *Tilia cordata*, *Valeriana officinalis*
Circulatory and blood system disorders	1	13	18	*Achillea millefolium*, *Allium sativum*, *Juniperus communis*

## Data Availability

All of the data supporting the reported results can be found in this article.

## Supplementary Materials

The following supporting information can be downloaded at: https://www.mdpi.com/article/10.3390/plants14010122/s1, Table S1: Comparative study methodologies and contexts for ethnobotanical research in the Bellino and Ubaye valleys.
