# Supplementary material for: Old Plants for New Food Products? The Diachronic Human Ecology of Wild Herbs in the Western Alps
Source: Plants (Basel). 2025 Jan 3;14(1):122. doi: 10.3390/plants14010122 (PMC11723087; doi:10.3390/plants14010122)
Supplement: Supplementary file 1 [file plants-14-00122-s001.zip › plants-3349572-supplementary.pdf]

Supplementary Information

Old Plants for New Food Products? The Diachronic Human Ecology of Wild Herbs in the Western Alps

Mousaab Alrhmoun [1,2], Aurora Romano [1], Naji Sulaiman [1,*] and Andrea Pieroni [1,3]

[1] University of Gastronomic Sciences, Piazza Vittorio Emanuele II 9, 12042 Pollenzo, Italy
[2] Faculty of Agricultural, Environmental and Food Sciences, Free University of Bolzano, Piazza Università 5, 39100 Bolzano, Italy
[3] Department of Medical Analysis, Tishk International University, Erbil 4001, Iraq

***Correspondence Author:** Naji Sulaiman
n.sulaiman@unisg.it

Table S1. Comparative study methodologies and contexts for ethnobotanical research in the Bellino and Ubaye valleys.

Methodology	Study 1: Varaita valley, Italy-Bellino valley Current study (2024)	Study 2: Varaita valley, Italy - Bellino and Ubaye valley, France (Pieroni and Giusti, 2009)	Study 3: Alpes de Haute Provence, Ubaye valley, France (Novaretti and Lemordant (published in 1990)
**Study Sites**	Bellino valley (Italy)	Varaita valley, Western Alps, Piedmont region	Ubaye valley, Alpes de Haute Provence
**Geographical Context**	Alpine pass connecting Italy and France; Occitan linguistic heritage	Varaita valley flowing from Monviso Mountain slopes	Geology with sedimentary calcareous rocks and siliceous formations supporting unique flora
**Altitude Range**	1,400 m (Ribiera) to 3,340 m (Mongioia)	Varaita valley from Monviso slopes	From plains-like Haute-Ubaye to alpine and subalpine zones
**Ethnographic Context**	Occitan-speaking communities with preserved cultural heritage	Occitan linguistic diversity, independent Escartons federation	Isolated community with a strong medicinal plant tradition
**Environmental Context**	Glacial valley, harsh winters, cool summers, unique fog patterns	Sub-littoral alpine climate, alpine vegetation, agro-pastoral economy	Complex geology and varied climate influencing flora diversity
**Data Collection**	Semi-structured interviews with residents, restaurateurs, experts	Semi-structured interviews with residents and restaurateurs	Literature review, field observations, and local interviews
**Participant Profile**	30 participants (10 men, 20 women, aged 30-85)	Local informants (n = 67) were asked about local food and medicinal plants	63 Ubayans, from 35 to 65 years
**Sampling Method**	Purposive sampling, prioritizing female elders for traditional knowledge	Purposive sampling of culturally knowledgeable residents	Purposive sampling of the knowledgeable people about medicinal plants.
**Fieldwork Methodology**	Interviews and fieldwork with experts on plant identification	Interviews and fieldwork with expert residents on plant identification	Fieldwork included mapping plant communities and habitat
**Comparison of cultural practices**	Italian participants' cultural practice	Cross-generational and historical comparisons	Differences noted across subregions within the valley
